# Natural Sesquiterpene Lactones Induce Oxidative Stress in *Leishmania mexicana*

**DOI:** 10.1155/2013/163404

**Published:** 2013-06-04

**Authors:** Patricia Barrera, Valeria P. Sülsen, Esteban Lozano, Mónica Rivera, María Florencia Beer, Carlos Tonn, Virginia S. Martino, Miguel A. Sosa

**Affiliations:** ^1^Instituto de Histología y Embriología “Dr. Mario H. Burgos” (IHEM-CONICET), Facultad de Ciencias Médicas, Universidad Nacional de Cuyo (UNCuyo), CC 56 (5500) Mendoza, Argentina; ^2^Instituto de Química y Metabolismo del Fármaco (IQUIMEFA) (UBA-CONICET), Cátedra de Farmacognosia, Facultad de Farmacia y Bioquímica, Universidad de Buenos Aires, Junín 956 2°P, 1113 Buenos Aires, Argentina; ^3^Instituto de Investigaciones en Tecnología Química (INTEQUI-CONICET), Facultad de Química, Bioquímica y Farmacia, Universidad Nacional de San Luis, 5700 San Luis, Argentina

## Abstract

Leishmaniasis is a worldwide parasitic disease, caused by monoflagellate parasites of the genus *Leishmania*. In the search for more effective agents against these parasites, the identification of molecular targets has been attempted to ensure the efficiency of drugs and to avoid collateral damages on the host's cells. In this work, we have investigated some of the mechanisms of action of a group of natural sesquiterpene lactones that are effective against *Leishmania mexicana mexicana* promastigotes. We first observed that the antiproliferative effect of mexicanin I (Mxc), dehydroleucodine (DhL), psilostachyin (Psi), and, at lesser extent, psilostachyin C (Psi C) is blocked by 1.5 mM reduced glutathione. The reducing agent was also able to reverse the early effect of the compounds, suggesting that lactones may react with intracellular sulfhydryl groups. Moreover, we have shown that all the sesquiterpene lactones, except Psi C, significantly decreased the endogenous concentration of glutathione within the parasite. Consistent with these findings, the active sesquiterpene lactones increased between 2.7 and 5.4 times the generation of ROS by parasites. These results indicate that the induction of oxidative stress is at least one of the mechanisms of action of DhL, Mxc, and Psi on parasites while Psi C would act by another mechanism.

## 1. Introduction

Leishmaniasis is a parasitic disease caused by flagellated parasites of the genus *Leishmania* and transmitted by phlebotomine sandflies. These parasites exhibit a heteroxenous life cycle, alternating between intracellular amastigotes in the mammalian cells and flagellate promastigotes in the vector.

Leishmaniasis affects about 12 million people worldwide and, according to the World Health Organization (WHO), 2 million of new cases occur annually and 350 million people are considered at risk of contracting leishmaniasis [1]. The clinical forms of the disease depend on the species of *Leishmania* involved and include local infections of the skin, subcutaneous tissue, and regional lymphatic nodes (cutaneous leishmaniasis); metastatic infections of the oronasal mucosa (mucocutaneous leishmaniasis); and disseminated infection involving visceral organs (visceral leishmaniasis) [2].

Leishmaniasis is distributed worldwide with foci of infection in Central and South America, Southern Europe, North and East Africa, the Middle East and India [3]. In Argentina, this parasitosis affects the northern region of the country with an incidence that has increased over the last two decades [4].

Current drugs used to treat leishmaniasis include pentavalent antimonials, pentamidine, and amphotericin B, which induce serious toxic effects on patients. Parasite resistance to these drugs has also been described. New formulations, such as liposomal amphotericin B and other drugs (miltefosine, paromomycin), have serious drawbacks such as parenteral route of administration, duration of the treatment, teratogenic effects, toxicity, and cost of treatment, which limit their use in endemic areas [5]. Therefore, there is an urgent need for novel candidates to treat this parasitic disease.

Sesquiterpene lactones, a group of natural compounds characteristic of the Asteraceae family, have been pointed out as good candidates for antiprotozoal therapy since many of them are active against trypanosomatids [6–8]. Moreover, we have previously described the trypanocidal and leishmanicidal activity of natural sesquiterpene lactones isolated from Argentinean Asteraceae species [9–16]. 

One of the most important aspects in antiprotozoal drug discovery is to determine the mechanism of action of the potential candidates and to identify the possible molecular targets upon which these compounds act. Among other mechanisms, it is presumed that sesquiterpene lactones could exert their leishmanicidal activity by the generation of an oxidative environment within the parasite [17, 18]. The particular defense mechanism against oxidative stress in trypanosomatids makes parasites susceptible to these kinds of compounds.

In this sense, the aim of the present work was to evaluate the possible effect of four bioactive sesquiterpene lactones: dehydroleucodine (DhL); mexicanin I (Mxc). psilostachyin (Psi), and psilostachyin C (Psi C) on the defense mechanism of *Leishmania mexicana mexicana* against oxidative stress.

## 2. Materials and Methods

### 2.1. Compounds

Mexicanin I (Mxc) was isolated from the aerial parts of *Gaillardia megapotamica* and dehydroleucodine (DhL) was isolated from *Artemisia douglasiana* as previously described [19]. Psilostachyin (Psi) and psilostachyin C (PsiC) have been isolated from *Ambrosia tenuifolia* and *A. scabra*, respectively [11, 13]. 

### 2.2. Parasites

Axenic cultures of *Leishmania mexicana mexicana *promastigotes were grown in Diamond's liquid medium (0.106 M NaCl, 29 mM KH_2_PO_4_, 23 mM K_2_HPO_4_, 12.5 g/L tryptone, 12.5 g/L tryptose, and 12.5 g/L yeast extract, adjusted to pH 7.2) supplemented with 75 *μ*M hemine, 75 IU/mL penicillin, 75 *μ*g/mL streptomycin, and 20% fetal bovine serum at 25°C. 

### 2.3. Treatments

*Leishmania mexicana mexicana *promastigotes (2 × 10^6^ parasites) were incubated with 0.5 *μ*g/mL of Mxc, Psi, or Psi C or 2.5 *μ*g/mL of DhL, at 25°C, either in the presence or in the absence of 1.5 mM glutathione (GSH). The concentrations used for each compound were those corresponding to each IC_50_, as previously determined (data not shown). Aliquots of the parasites were collected every 24 h and counted in a Neubauer hemocytometer [16]. In other experiments, parasites were preincubated with the compounds for 30 min and the reducing agent was then added. Alternatively, the lactones were withdrawn after incubation for 1 h and before adding GSH. Controls were carried out in the presence of DMSO (less than 0.05%) which was used to dissolve the compounds.

### 2.4. Measurement of ROS

The fluorescent probe, H_2_DCFDA, was used to measure the intracellular generation of ROS, according to Duranteau et al. [20]. Briefly, parasites (1 × 10^6^ cells) were previously treated with the lactones (10 *μ*g/mL of each sesquiterpene lactone for 3 h) and then incubated with 10 *μ*M of the probe for 1 h at room temperature in the dark. The fluorescence intensity of H_2_DCFDA was measured at 507 nm excitation and 538 nm emission wavelengths. To validate the assay, generation of ROS by 4 mM H_2_O_2_ was used as a positive control. 

### 2.5. Measurement of Reduced Glutathione

Endogenous GSH was measured in parasite lysates by using 5,5′-dithiobis-2-nitrobenzoic acid (DTNB), according to Beutler et al. [21]. Briefly, parasites (1 × 10^7^ cells/mL) were previously incubated with the lactones (10 *μ*g/mL) for 3 h at 25°C then pelleted, lysed with 200 *μ*L lysis solution (10% EDTA, 0.5% Triton X-100 in bidistilled water) during 30 min, and centrifuged at 12,000 ×g. Supernatants were mixed with 300 *μ*L of solution P (0.2 M HPO_3_, 5 mM EDTA, and 5.1 M NaCl) and centrifuged again at 12,000 ×g. Supernatants were mixed with 800 *μ*L of 0.3 M Na_2_HPO_4_, and 200 *μ*L of DTNB (in 1% sodium citrate). Absorbances were then measured in a spectrophotometer at 412 nm, and the concentration of GSH was derived from a standard curve.

### 2.6. Statistical Analysis

Results are presented as mean ± SD. The level of statistical significance was determined by using one-way analysis of variance (ANOVA) followed by Dunnett's multiple comparisons test. 

## 3. Results and Discussion

**Figure 1:**
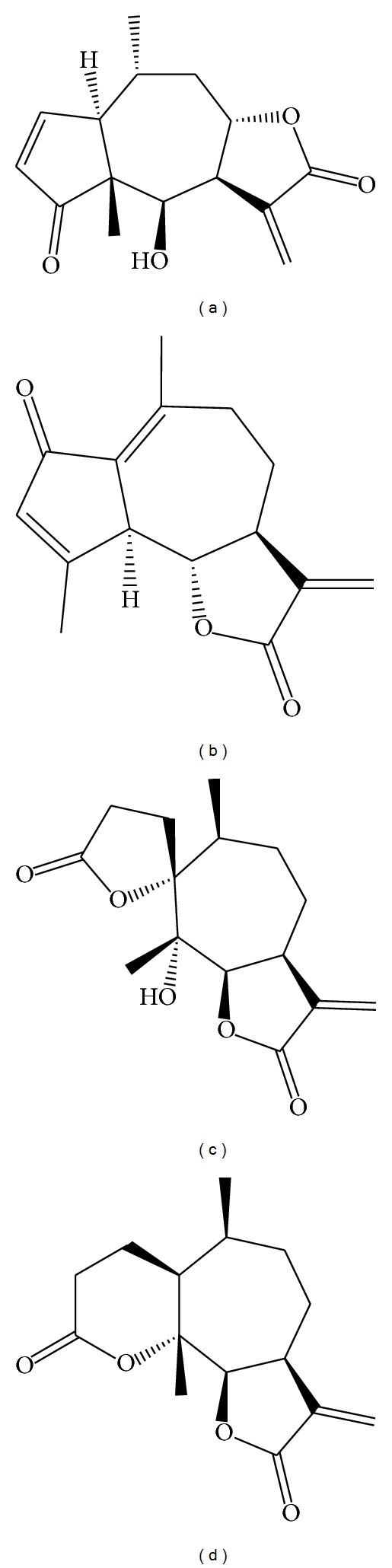
Chemical structures of the sesquiterpene lactone: mexicanin I (a), dehydroleucodine (b), psilostachyin (c), and psilostachyin C (d).

We had previously reported the antileishmanial activity of Mxc, DhL, Psi, and Psi C (Figure 1) [9–16]. The common functional group *α*-methylene-*γ*-lactone present in the sesquiterpene lactones is believed to be responsible for their antiprotozoal activity. However, the presence of other alkylating groups such as *α*,*β*-unsaturated cyclopentenones and other factors, such as lipophilicity, molecular geometry, and chemical environment, may also influence their bioactivity [22].

**Figure 2:**
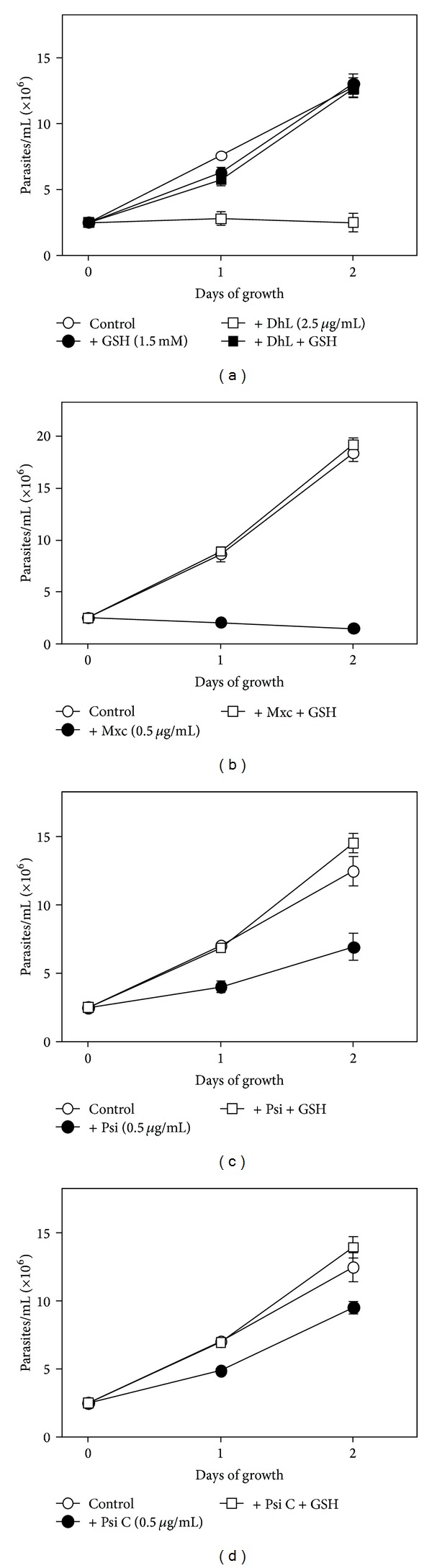
The effect of sesquiterpene lactones on the growth of *L. mexicana mexicana *is blocked by adding of GSH. Parasites were incubated with the lactones; dehydroleucodine (DhL) (a), mexicanin I (Mxc) (b), psilostachyin (Psi) (c), or psilostachyin C (Psi C) (d) in the presence or in the absence of 1.5 mM glutathione (GSH), as indicated in the figure. Parasite counts were done daily. Glutathione alone did not affect the parasite growth (a).

**Figure 3:**
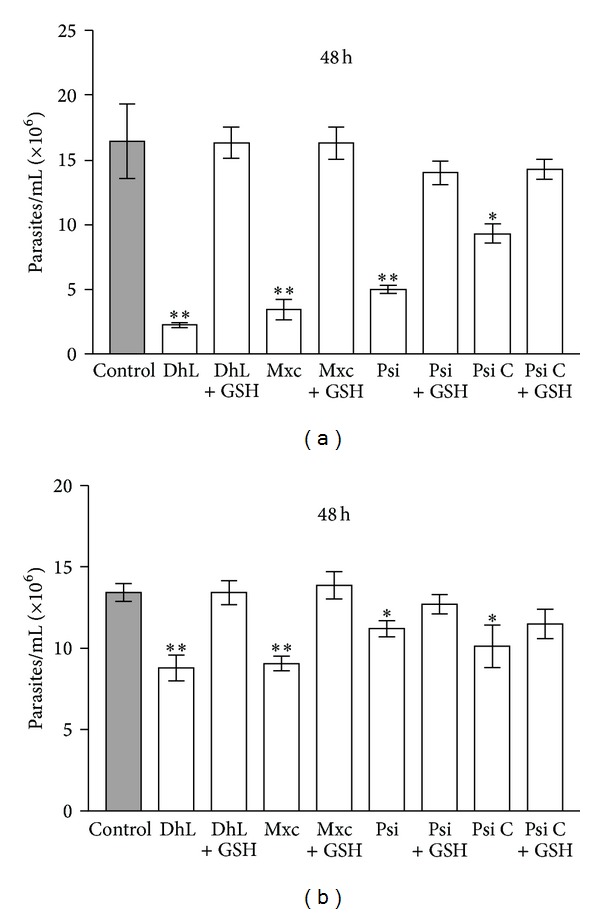
The effect of glutathione (GSH) on the number of parasites preincubated with 0.5 *μ*g/mL mexicanin I (Mxc), psilostachyin (Psi) or psilostachyin C (Psi C) or with 2.5 *μ*g/mL of dehydroleucodine (DhL), for 30 min (a) or preincubated 1 h with the lactones and followed by withdrawal of the compounds before adding the reducing agent (b). Bars represent the means of parasite concentration ± SD from three independent experiments. (∗∗) and (∗) indicate significant differences with the control (*P* < 0.01 and *P* < 0.05 resp.).

**Figure 4:**
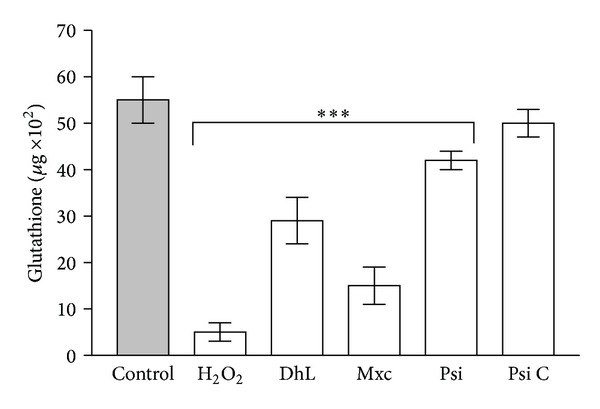
Concentration of endogenous glutathione in the parasites after treatment with 10 *μ*g/mL dehydroleucodine (DhL), mexicanin I (Mxc), psilostachyin (Psi), or psilostachyin C (Psi C), as described in materials and methods. (∗∗∗): significant differences with the control (*P* < 0.02). H_2_O_2_ (5 mM) was used as positive control.

In this work we have corroborated the antiproliferative effect of the four lactones on *L. mexicana mexicana* promastigotes and we have demonstrated that this effect was blocked by 1.5 mM GSH (Figure 2). As these lactones are nonpolar molecules they could easily pass through the parasite's plasmalemma. The blocking effect of GSH might be due to the transformation of the compounds into derivatives unable to traverse the plasmalemma. However, it is more likely that the compounds interfere with the intracellular concentration of GSH, as the antiproliferative effect of lactones can be reversed by GSH when the reducing agent is added 30 min after incubation with the compounds or 1 h after incubation followed by withdrawal of the lactones (Figure 3). In addition, it was observed that DhL, Mxc, and Psi, but not Psi C, reduced the concentration of endogenous GSH (Figure 4). 

**Figure 5:**
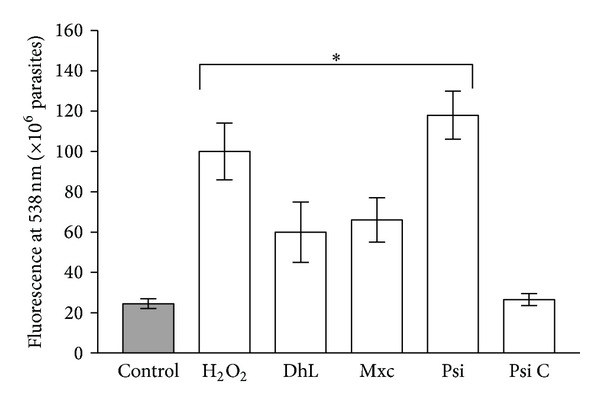
Generation of ROS by the parasites after treatment with 10 *μ*g/mL of mexicanin I (Mxc), dehydroleucodine (DhL), psilostachyin (Psi), and psilostachyin C (Psi C). Values are expressed as units of fluorescence emitted by the probe at 538 nm. Bars represent the means of fluorescence ± SD from three independent experiments. (∗): significant differences with the control (*P* < 0.05).

On the other hand, treatment with DhL, Mxc, or Psi, but not Psi C, induced a significant increase of ROS in *L. mexicana* promastigotes (Figure 5).

The generation of free radicals in *Leishmania* by the sesquiterpene lactones would be deleterious for trypanosomatids, as the regulation of oxidative stress is crucial for parasite survival. It is known that sesquiterpene lactones react with sulfhydryl groups by the Michael-type addition and therefore could act by inhibiting the activity of enzymes that are vital against oxidative stress (e.g., trypanothione reductase) [17]. This situation could lead to an increase in the level of reactive oxygen species and to parasite damage via the generation of an oxidative burst by a deregulation of the redox balance within the parasite [23]. However, a direct interaction of the compounds with GSH or trypanothione should not be ruled out. 

The decrease in the concentration of glutathione within the parasites induced by the sesquiterpene lactones Mxc, DhL, and Psi would lead to an enhancement in the production of reactive oxygen species. These results are in accordance with ROS production and the *in vitro* leishmanicidal activity, with psilostachyin C being the less active compound against *L. mexicana*. Given that sesquiterpene lactones can also induce GSH depletion and ROS generation in certain mammalian cells (e.g., tumor cells) [24], these compounds should be improved before use as therapeutic agents against *Leishmania.*

One vital step in the process of drug development is the identification of the molecular target/s of such drugs. Taking into consideration the data obtained, we can suggest that Mxc, DhL, and Psi were able to affect the defense mechanism against oxidative stress in *L. mexicana*. This mechanism could be related to inhibition of key enzymes that maintain redox balance in the parasite. 

This study must be complemented by further investigations on amastigotes forms of *Leishmania* and on *in vivo* models of leishmaniasis.
